# An Unexpected Cause of Eye Irritation: A Case of Zoonotic Ocular Onchocerciasis

**DOI:** 10.1155/2013/504749

**Published:** 2013-12-03

**Authors:** Abhishek Biswas, Mohamed H. Yassin

**Affiliations:** ^1^Department of Internal Medicine, University of Pittsburgh Medical Center East, 2775 Mosside Boulevard, Monroeville, PA 15146, USA; ^2^Infectious Diseases Division, University of Pittsburgh School of Medicine, Pittsburgh, PA 15213, USA

## Abstract

A 19-year-old male residing in Pittsburgh presented with irritation and watering from his right eye and was diagnosed to have a right subconjunctival nodule. Surgical excision revealed both dead and living worms and histopathological staining of the worms confirmed these to be zoonotic species of *Onchocerca.* The morphologic characteristics of the worm suggest it to be either *O. lupi* or *O. lienalis* which were first detected in wolves and cattle, respectively. Mystery remains as to the mode of transmission and the hosts for this parasite in this part of the United States. This case adds to the growing number of cases of zoonotic ocular onchocerciasis reported from all over the world.

## 1. Introduction

Onchocerciasis is the world's second-leading infectious cause of blindness and is estimated to account for blindness in half a million people. It is transmitted by the bite of the *Simulium* fly which transmits the infective stage larva into the skin. The vast majority of infections occur in sub-Saharan Africa and is rarely seen in the US. We report an interesting case of a young male residing in Pittsburgh who presented with a sub-conjunctival nodule, biopsy of which revealed both dead and living worms. Further studies suggested this to be either *O. lupi* or *O. lienalis*, which are species of *Onchocerca* that usually infect animals. There are twenty-one published reports of human infections with zoonotic species of *Onchocerca*. This case of zoonotic ocular infection with *Onchocerca* sp. is the third reported case from the United States and is unique since zoonotic onchocerciasis has not been reported from the East coast till date.

## 2. Case Report

A 19-year-old Egyptian male studying and living in Pittsburgh, PA, USA, for 18 months was evaluated for a month-long irritation and watering of the right eye without any visual loss. He had been seen by an ophthalmologist and was prescribed topical steroids for possible allergic conjunctivitis, which did not resolve his irritation.

**Figure 1:**
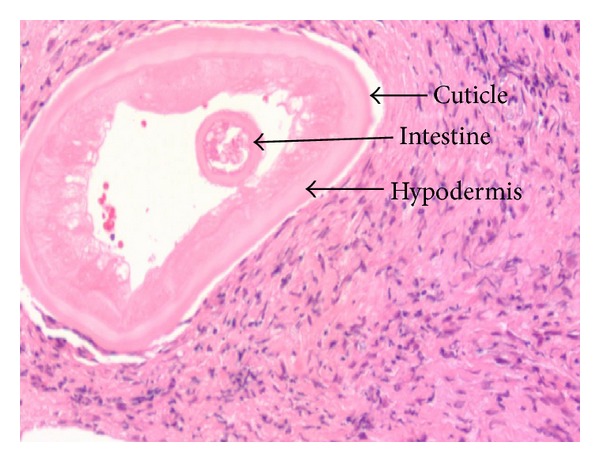
Transverse section of the adult worm (H&E stained) showing the internal organs.

**Figure 2:**
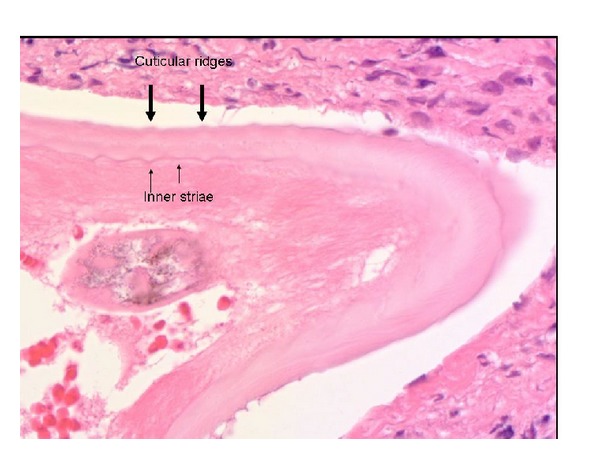
Higher magnification of the same worm shows arrangement of the cuticle with external ridges and internal striae. There are two striae for each interridge—one in between two ridges and one striae immediately under each ridge.

The patient's recent travels included a trip to New Delhi, India, and Siwa (an oasis in the West of Egypt) more than a year prior to this presentation. He has never had any pets such as dogs and cats. Review of systems as well as general examination was unremarkable. Ophthalmologic examination revealed a subconjunctival nodule (1.5 cm × 1 cm) in the medial aspect of the right eye. The rest of the eye examination was unremarkable including the retina and the posterior chamber. He underwent surgical exploration of the nodule under local anesthesia, which revealed 12 dead and living worm fragments, each about 1 cm in length and 0.2 to 0.3 mm in thickness. Serology showed a positive IgG4 for filariasis. Complete blood count was normal and peripheral blood smear was negative for eosinophilia or microfilaria. Histopathological examination revealed adult worms that were confirmed to be *Onchocerca* sp. based on specific morphologic features. The worms were immediately placed in saline and subsequently preserved in ethanol and glycerol. Histological examination of an individual worm after staining with hematoxylin and eosin indicated that it was a female adult *Onchocerca* (Figures 1 and 2). There was no evidence of microfilaria (mf) in the uteri. The Centers for Diseases Control and Prevention (CDC) confirmed the diagnosis. The patient was treated with Ivermectin (100 mg/kg) once and has had no further recurrence of his symptoms after two years of followup.

## 3. Discussion

*Onchocerca* is a filarial nematode that is known to cause multiple skin manifestations. However, the most important presentation of Onchocerciasis is its ophthalmologic manifestations and which is also commonly referred to as “river blindness” [1]. Endemic ocular Onchocerciasis could be in the form of a nodule (as in this case), keratitis (anterior chamber), chorioretinitis (posterior chamber), or optic atrophy [2]. On the other hand, zoonotic Onchocerciasis is being reported in different parts of the world which includes a few cases from the United States as well. Our knowledge about this entity is limited because of its rarity.

Crucial to making this diagnosis is to identify the worm on histopathological examination. Speciation of such worms is often not possible because of improper preservation or a lack of an intact worm structure. We will briefly present the main diagnostic features of the adult worm. A multilayered cuticle forms the outer thick covering of a female adult *Onchocerca*. Annular ridges characterize the external layer and transverse striae are found on the internal layer. However, this identifying feature is less prominent in the male worm. Thus, knowledge of these micromorphologic features is critical to identifying these worms [3]. The worm mentioned in this report was a nongravid adult worm having an external cuticle with low rounded ridges running around it and one internal stria under each ridge and one in between (Figure 2). *O. volvulus*, *O. lupi*, and *O. lienalis* (zoonotic *Onchocerca* found in cattle) have similar morphology, that is, two striae per ridge (one under and one between). Additional molecular studies can definitively distinguish between these three species [4].

Table 1 enumerates the different species of *Onchocerca* that have been known to cause human disease worldwide and also highlights the major identifying points for the adult worm of each species [5–8]. As mentioned before, both *O. volvulus* and *O. lienalis* have two striae for every interridge similar to the arrangement for *O. lupi*.

*O. volvulus* infection is not prevalent in the USA and mostly occurs in West Africa, Central and South America. Although our subject's travel history to Egypt and India raises the possibility of contracting the disease during travel, Onchocerciasis has not been known to be endemic in those two countries. Based on the morphology, we feel that it belongs to the species *O. lupi* or *O. lienalis*.

Zoonotic Onchocerciasis has been reported to occur from the North American continent as well as from Europe. There have been recent reports of zoonotic Onchocerciasis from the West Coast of the United States [9]. There have also been reports of cryptic infections with *O. lupi* presenting with sub-conjunctival nodules from Europe [10]. A review of the literature reveals eight reported cases of ocular Onchocerciasis from around the world. Four of these cases had sub-conjunctival nodules containing the adult worm and one other patient had the worm located in the tendon of the globe. In the remaining three patients, the worm was retrieved from the anterior chamber of the eye [11]. *O. lupi* seems to be the most common zoonotic species affecting the eye [5]; other species that have been reported with human infections include *O. cervicalis*, *O. gutturosa*, and *O. dewittei* [9]. These aberrant infections have been known to occur in joints, soft tissues of the head, foot, abdomen, shoulder, and cervical spine [5].

There are limited pharmacological choices for the management of ocular Onchocerciasis. ivermectin is the recommended treatment for microfilaria but has no effect on the adult worms [11]. For Onchocerciasis affecting people in endemic areas, ivermectin may be given annually for the lifetime of the adult worm (15 years). But for our patient we felt that, this being a zoonotic localized disease and the worm being totally removed by the ophthalmologist, no further treatment would be necessary [12]. The two-year followup confirmed that the patient had no recurrence of his illness.

Thus we conclude that zoonotic ocular Onchocerciasis can present with a sub-conjunctival nodule in humans and it should be considered as a differential diagnosis of parasites detected in the ocular structures. The exact mode of transmission is unclear at this time, as is the prevalence of this infestation among cattle or wildlife in this part of the United States. Further information on the epidemiology of the disease among the animals population would be needed before we can understand the risk factors for transmission of the disease to humans.

## Figures and Tables

**Table 1:** Morphologic features of zoonotic *Onchocerca* species and their chief identifying feature.

*Onchocerca* sp.	Identifying characteristics
*O. guttorosa *(cattle)	Four striae per interridge
*O. cervicalis *(horses)	Three to four striae per inter-ridge
*O. lupi *(wolves, cats)	Two internal striae per every inter-ridge
*O. jakutensis *(European deer)	Three to four elongated striae per inter-ridge
*O. dewittei japonica *(wild boar)	Marked external transverse with no inner striae [13].
